# Unique immune and other responses of human nasal epithelial cells infected with H5N1 avian influenza virus compared to seasonal human influenza A and B viruses

**DOI:** 10.1080/22221751.2025.2484330

**Published:** 2025-03-24

**Authors:** Kai Sen Tan, Jing Liu, Anand Kumar Andiappan, Zhe Zhang Ryan Lew, Ting Ting He, Hsiao Hui Ong, Douglas Jie Wen Tay, Zhen Qin Aw, Bowen Yi, Arfah Mohd Fauzi, Thinesshwary Yogarajah, Lee Ching Pei Carmen, Justin Jang Hann Chu, Vincent T. Chow, Mookkan Prabakaran, De-Yun Wang

**Affiliations:** a Infectious Diseases Translational Research Programme and Department of Microbiology and Immunology, Yong Loo Lin School of Medicine, National University of Singapore, Singapore, Singapore; b Biosafety Level 3 Core Facility, Yong Loo Lin School of Medicine, National University of Singapore, Singapore, Singapore; c Infectious Diseases Translational Research Programme and Department of Otolaryngology, Yong Loo Lin School of Medicine, National University of Singapore, Singapore, Singapore; d Singapore Immunology Network (SIgN), Agency for Science, Technology and Research (A*STAR), Singapore, Singapore; e Collaborative and Translation Unit for HFMD, Institute of Molecular and Cell Biology, A*STAR, Singapore, Singapore; f Temasek Life Sciences Laboratory, Singapore, Singapore

**Keywords:** Influenza, highly pathogenic avian influenza, H5N1, upper airway, human nasal epithelial cells, host pathogen interactions, molecular responses

## Abstract

Highly pathogenic avian influenza (HPAI) virus (e.g. H5N1) infects the lower airway to cause severe infections, and constitute a prime candidate for the emergence of disease X. The nasal epithelium is the primary portal of entry for respiratory pathogens, serving as the airway's physical and immune barrier. While HPAI virus predominantly infects the lower airway, not much is known about its interactions with the nasal epithelium. Hence, we sought to elucidate and compare the differential responses of the nasal epithelium against HPAI infection that may contribute to its pathology, and to identify critical response markers. We infected human nasal epithelial cells (hNECs) cultured at the air–liquid interface from multiple healthy donors with clinical isolates of major human seasonal influenza viruses (H1N1, H3N2, influenza B) and HPAI H5N1. The infected cells were subjected to virologic, transcriptomic and secretory protein analyses. While less adapted to infecting the nasal epithelium, HPAI H5N1 elicited unique host responses unlike seasonal influenza. Interestingly, H5N1 infection of hNECs induced responses indicative of subdued antiviral activity (e.g. reduced expression of IFNβ, and inflammasome mediators, IL-1α and IL-1β); decreased wound healing; suppressed re-epithelialization; compromised epithelial barrier integrity; diminished responses to oxidative stress; and increased transmembrane solute and ion carrier gene expression. These unique molecular changes in response to H5N1 infection may represent potential targets for enhancing diagnostic and therapeutic strategies for better surveillance and management of HPAI infection in humans.

## Introduction

Influenza viruses remain a global health challenge, and even more so after the world has transitioned from the COVID-19 pandemic [1]. The usual annual influenza seasons are typically caused by human-adapted seasonal influenza A (H1N1 and H3N2) and influenza B (B/Victoria and B/Yamagata) virus strains (seasonal influenza). In addition, zoonotic influenza viruses, particularly the highly pathogenic avian influenza (HPAI) viruses such as H5N1 and H7N9 [2,3], constantly threaten public health with sporadic, intermittent outbreaks, including recent Southeast Asian outbreaks in 2023 [4,5] and the US outbreaks in cattle spilling over to humans in 2024 [6,7]. The outbreak of bovine H5N1 (clade 2.3.4.5b) associated with some human infections is of great epidemiologic significance as it shows that this H5N1 is now capable of transmission within mammalian hosts [8]. Both seasonal and avian influenza viruses have the potential to cause severe infection which may lead to pneumonia, acute respiratory distress syndrome (ARDS), and even death [9]. Different seasonal influenza virus subtypes and strains possess varying virulence to cause a spectrum of disease severity. In contrast, avian influenza virus infections in humans incur a greater risk of causing severe disease and complications, due to its preference to infect the lower airway [9–11]. Despite the preference of avian influenza for the lower airway, the upper airway remains a vital primary contact site during the initial infection and is currently underexplored [11]. Moreover, investigating the infectivity and responses of the upper airway is crucial to better understand transmissibility of current and emerging respiratory viruses, e.g. utilizing primary airway cultures for assessing SARS-CoV-2 transmission during the COVID-19 pandemic [12]. In view of the disquieting shift in H5N1 transmission to cattle, where there may be a potential change in preference to target upper airway cells [8,13], it is vital to investigate how HPAI viruses like H5N1 interact with the upper airway in order to enhance the surveillance of future HPAIs transmission. Therefore, understanding these vital interactions between the upper airway and influenza viruses is crucial for unravelling the traits of HPAI infections which distinguish them from seasonal influenza infections.

The human nasal epithelium is the first line of defense against incoming pathogens in the upper airway[14,15]. The pseudostratified columnar epithelium utilizes mechanical (cilia and mucus) and immunological (cytokine and chemokine secretion) means against invading pathogens to facilitate their clearance [16]. On the other hand, influenza viruses evolve their own immune evasive strategies, including interferon antagonism and translational repression to facilitate their replication in the airway, culminating in acute airway inflammation and disease manifestations [17-19]. Avian influenza viruses are not as adapted to infect the upper airway and more frequently cause lower airway inflammation and pneumonia with greater disease severity [11,20]. Hence, the current focus with avian influenza is on management strategies to target the lower airway where the disease manifests. However, it is of clinical significance to also elucidate the detailed interactions of avian influenza (especially HPAI) viruses with the upper airway epithelium to unravel the mechanism that underpin disease pathogenesis and manifestations. Such investigations may discover potentially useful markers or targets to improve early diagnostic and intervention strategies, as well as early threat assessments for avian influenza infections and their potential for transmission to humans.

Therefore, the aim of this study was to compare and contrast the molecular changes in the upper airway epithelium in response to avian versus seasonal influenza viruses, by using representative influenza strains isolated from clinical infections. To achieve this, we utilized *in vitro* differentiated, air–liquid interface (ALI) culture of human nasal epithelial cells (hNECs) to elucidate the virologic, transcriptomic and cytokine alterations following influenza infections [14]. We utilized clinical isolates of seasonal influenza subtypes (H1N1, H3N2 and B/Victoria) to establish common responses to seasonal influenza infections. These responses were compared against infection with a clinical isolate of H5N1 HPAI to determine differential patterns of responses of upper airway infections. This comparative analysis led to the identification of critical molecular factors that contribute to differential pathogenesis of HPAI, which may potentially be harnessed to establish patterns for threat assessment, diagnostic and treatment purposes.

## Materials and methods

### Derivation of human nasal epithelial stem/progenitor cells (hNESPCs) and in vitro differentiation of hNECs

Approval to conduct this study was obtained from the National Healthcare Group Domain-Specific Board of Singapore (DSRB Ref: D/11/228) and Institutional Review Board of the National University of Singapore (IRB Ref: 13-509). Written consent was obtained from donors prior to the collection of the nasal tissue biopsies from subjects who were free of symptoms of upper respiratory tract infection. The hNESPCs were isolated and enriched from the tissue biopsies according to a previously standardized protocol [14,21], which normalized the hNESPCs to a baseline state that differentiates into hNECs in a healthy state [21]. Following enrichment, the hNESPCs were expanded further and subjected to ALI culture in transwells for *in vitro* differentiation as described previously [14,21]. Briefly, primary cells were subjected to selection of hNESPCs which were enriched and expanded with Dulbecco's modified Eagle medium: nutrient mixture F-12 (DMEM/F12; Gibco-Invitrogen, Waltham, MA, USA) containing 10 ng/mL of human epithelial growth factor (EGF; Gibco-Invitrogen, Waltham, MA, USA), 5 μg/mL of insulin (Sigma, St Louis, MO, USA), 0.1 nM of cholera toxin (Sigma-Aldrich, St Louis, MO, USA), 0.5 μg/mL of hydrocortisone (Sigma-Aldrich, St Louis, MO, USA), 2 nM of 3, 3’, 5-triiodo-L-thyronine (T3; Sigma-Aldrich, St Louis, MO, USA), 10 μL/mL of N-2 supplement (Gibco-Invitrogen, Waltham, MA, USA) and 100 IU/mL of antibiotic-antimycotic (Gibco-Invitrogen, Waltham, MA, USA). The expanded hNESPCs were then transferred onto 12-well 0.4 μm transwell inserts (Corning, Corning, NY, USA). Primary human bronchial epithelial cells (hBECs) (PromoCell, Heidelberg, Germany) were expanded in PneumaCult-Ex Plus medium (Stemcell Technologies, Vancouver, Canada) supplemented with 100 IU/mL antibiotic-antimycotic (Gibco-Invitrogen, Waltham, MA, USA), 1 μM A83-01 (Stemcell Technologies, Vancouver, Canada), 5 μM Y-27632 (Stemcell Technologies, Vancouver, Canada) and 3 μM isoprenaline hydrochloride (MedChemExpress, NJ, USA). Once confluent, growth medium was discarded, and 700 μL of PneumaCult™-ALI medium with inducer supplements (Stemcell Technologies, Vancouver, Canada) was added to the basal chamber to establish ALI conditions for culture of both hNECs and hBECs. The cells were cultured in ALI for 4 weeks, with change of medium every 2–3 days. After 3–4 weeks of differentiation, hNECs from a total of 7 donors were then subjected to seasonal and avian influenza virus infection. The hBECs were subjected to selected infection experiments to validate the findings of hNECs infection.

### Infection of fully differentiated hNECs with human seasonal and avian influenza virus strains

The human seasonal influenza A and B strains used in this study were isolated from outpatients in Singapore, i.e. A/Singapore/G2-25.1/2014 (H1N1) [22], A/Singapore/CDC-204/2012 (H3N2) [23], and B/Singapore/G2-14.1/2014 (Victoria lineage), or BVic [22]. These three strains were propagated by embryonated egg culture and used for infection of hNECs and RNA sequencing (RNAseq) analysis. Another H3N2 strain (A/Aichi/2/1968) was used for validation. The main avian influenza strain used in this study for hNECs infection and RNAseq is a clinical isolate derived from a fatal case of H5N1 influenza, i.e. A/Indonesia/CDC1031/2007 (H5N1), obtained from the Ministry of Health, Republic of Indonesia [24]. In addition, a H5N1 avian isolate i.e. A/mallard/Wisconsin/2576/2009 (H5N1) was also used to validate certain findings. Fully differentiated hNECs were washed with 1x DPBS before infection with the respective influenza viruses (H1N1, H3N2, BVic, H5N1) at a multiplicity of infection (MOI) of 0.1. The hNECs were also infected with H5N1 at MOI of 1.0. Mock infections with medium only served as controls matched for each collection time-point. The infected cultures were incubated for 1 hr at 35°C. The viral inoculum was then removed, and the hNECs were incubated at 35°C. At 8, 24 and 48 h post infection (hpi), the control and infected hNECs were subsequently harvested for their apical wash and RNA samples.

### Viral plaque assay

At each infection time-points, 150 µL of 1x DPBS was added in the apical chamber and incubated for 10 min at 35°C to recover progeny viruses as the apical wash. The plaque assay for viral quantification was performed using overnight cultures of Madin-Darby canine kidney (MDCK) cells (ATCC, Manassas, VA, USA) at 85–95% confluence in 24-well plates. The MDCK cells were incubated with 100 µL from each serial dilutions (from 10^−1^ to 10^−6^) of apical washes at 35°C for 1 hr, with rocking every 15 min to ensure equal viral distribution. The inoculum was then removed, and replaced with 1 mL of Avicel overlay (FMC Biopolymer, Philadelphia, PA, USA), and incubated at 35°C for 65–72 hr. The Avicel overlay was then removed, and cells were fixed with 4% formaldehyde in 1× PBS for 1 hr. Formaldehyde was removed, and cells were washed with 1× PBS prior to staining with 1% crystal violet for 15 min before washing the stain away. The plaque-forming units (PFU) were calculated as follows: (number of plaques × dilution factor)/0.1 mL = PFU/mL.

### Total RNA extraction and reverse transcription-quantitative PCR

After collection of apical wash at each time-point, the hNECs were lysed using RNA lysis buffer. Total RNA was then extracted from the lysate using mirVana miRNA isolation kit (Life Technologies, Grand Island, NY, USA). The extracted total RNA was subjected to Nanodrop analysis to first ensure the RNA quality, before being submitted for RNAseq analysis. Each RNA sample (500 ng) was subjected to cDNA synthesis using Maxima first-strand cDNA synthesis kit (ThermoScientific, Pittsburgh PA, USA). Quantitative PCR (qPCR) analysis was then performed to evaluate the transcriptional levels of selected host response genes using pre-designed primers (Sigma-Aldrich, St Louis, MO, USA) [15]. Each qPCR reaction was performed in duplicate using GoTaq-qPCR Master Mix kit (Promega, CA, USA), and relative gene expression was calculated using the comparative formula of 2^−ΔΔCt^ normalized to the housekeeping gene PGK1.

### Library preparation for RNAseq

All human RNAs were analysed on Agilent Bioanalyzer (Agilent, Santa Clara, CA, USA) or Perkin Elmer Labchip GX system (Perkin Elmer, Waltham, MA, USA) for quality assessment with RNA Integrity Number (RIN) or RNA Quality score range from 6.8-9.7 and median of 9.0. cDNA libraries were prepared using 2 ng of total RNA and 1ul of a 1:50,000 dilution of ERCC RNA Spike in Controls (Ambion® Thermo Fisher Scientific, Waltham, MA, USA) using SMARTSeq v2 protocol [25] except for the following modifications: 1. Use of 20 µM TSO, 2. Use of 250pg of cDNA with 1/5 reaction of Illumina Nextera XT kit (Illumina, San Diego, CA, USA). The length distribution of the cDNA libraries was monitored using DNA High Sensitivity Reagent Kit on the Perkin Elmer Labchip GX system (Perkin Elmer, Waltham, MA, USA). Sixteen samples were subjected to an indexed PE sequencing run of 2x 51 cycles on an Illumina HiSeq 2000 (16 samples/lane) and 65 samples to an indexed PE sequencing run of 2 × 151 cycles on an Illumina HiSeq 4000 (30 samples/lane).

### RNAseq analysis

FASTQ files were mapped to the human genome build hg38 using STAR. Gene counts were computed using featureCounts (part of the Subread package) using annotations from GENCODE version 26. Differential gene expression analysis between infected sample and the respective time-matched controls was performed using edgeR in a paired fashion under R version 3.3.3. Multiple testing correction was performed using the method of Benjamini and Hochberg and genes with *p* values (False Discovery Rate; or FDR) less than 0.05 were deemed to be significantly differentially expressed genes (DEGs).

### Gene set enrichment analysis

Gene set enrichment analysis using data from Gene Ontology (GO) was performed using the Bioconductor package topGO. The analysis based on the Reactome Pathway database was performed using the Bioconductor package ReactomePA. Both analyses were run in R version 3.3.3 using multiple testing corrected significant DEGs.

### Immunofluorescence staining

For microscopy experiments, hNECs were plated directly into Nunc Lab-Tek II Chamber slide (Thermo Fisher Scientific, Waltham, MA, USA) and then infected with either media-only control or 0.1 MOI of Avian H5N1 influenza (A/Indonesia/CDC1031/2007). At 48hpi, the cells were washed twice with DPBS and fixed with 4% formaldehyde for 10 min at room temperature (RT), followed by two washes of DPBS. After permeabilization with 0.1% Triton X-100 for 10 min at RT, the cells were blocked with 1% bovine serum albumin (BSA) in PBS (w/v) for 30 min at RT, and incubated with H5 haemagglutinin (HA)-specific mouse monoclonal antibody 2D9 (1:200 dilution) [26], epithelial markers rabbit monoclonal βIV-tubulin (1:400, ab179504, Abcam, Cambridge, UK), rabbit polyclonal MUC5AC (1:200, sc-20118, Santa Cruz Biotechnology, Santa Cruz, CA, USA) or rabbit monoclonal P63 (1:50, ab124762, Abcam, Cambridge, UK). After incubation, the cells were washed three times with DPBS, and incubated with the respective secondary anti-rabbit/mouse antibodies (Dako, Glostrup, Denmark) for 1 h RT, followed by another three times of washing. The cells were then mounted using ProLong AntiFade reagent with DAPI and covered with microslides. Cells were observed with a Leica SP8 laser scanning confocal microscope (Leica Microsystems, Mannheim, Germany). For generation of ALI-cross section staining, hNECs infected with Avian H5N1 influenza (A/mallard/Wisconsin/2576/2009) were fixed as per above, then embedded and sectioned as previously described [27]. Sectioned hNEC were blocked with 10% normal goat serum (50062Z, Invitrogen, USA), and stained with the following primary antibodies: H5 HA-specific mouse monoclonal antibody 3B1 (1:10) [28], epithelial markers rabbit monoclonal MUC5AC (1:400, ab198294, Abcam, Cambridge, UK), rabbit monoclonal α-tubulin (1:1000, ab179484, Abcam, Cambridge, UK), or mouse monoclonal P63 (1:50, ab735, Abcam, Cambridge, UK). The sections were then washed and stained with secondary anti-rabbit/mouse antibodies (1:500, Invitrogen, Waltham, MA, USA). For co-staining of H5 HA with epithelial markers raised from the same species, sequential sections were used, and the same regions were overlapped upon visualization. Sections were then washed and mounted with DAPI ProLong^TM^ Gold AntiFade Mountant with DAPI stain (Invitrogen, Waltham, MA, USA) and visualized with a Nikon Ni-U microscope and captured with a DS-Ri2 camera (Nikon, Tokyo, Japan).

### LUMINEX assay

To validate the functional responses of hNECs against different influenza infections, LUMINEX assay was performed on the apical wash supernatant from control and infected hNECs at 48 hpi. A custom LUMINEX panel for 17 cytokines (R&D Systems, Minneapolis, MN, USA) was employed to quantify levels of secretory factors indicative of viral infection, and proteins in pathways selected from RNAseq analysis. The readings of each tested factor was calculated according to the standard curve of the corresponding factor. The 1x DPBS served as the blank control.

### Peruvoside treatment

The efficacy of the ion channel modulatory drug peruvoside was evaluated against infections with different seasonal and avian influenza virus strains. Fully differentiated hNECs were infected with A/Aichi/2/1968 (H3N2) strain at MOI of 0.1. Fully differentiated hNECs and hBECs were infected with A/mallard/Wisconsin/2576/2009 (H5N1) strain at MOI of 0.1. The hNECs and hBECs were washed with 1x DPBS, and incubated with the virus at 35°C for 1 hr. The virus inoculum and basal medium were removed, and replaced with 25 and 350 µL of media containing peruvoside at the apical and basal chambers, respectively. The peruvoside concentrations tested were 20, 60 and 100 nM, with DMSO serving the untreated control. The treated cells were then incubated at 35°C for 48 hrs. Finally, the apical wash in 1x DPBS was collected from each well and subjected to virus plaque assay to determine the viral titre.

### Statistical analysis

Statistical analyses of experimental results were performed using GraphPad PRISM 8. The individual statistical tests are described in the respective figure legends.

## Results

### H5N1 avian influenza virus replicates less efficiently in the human nasal epithelium

**Figure 1.**
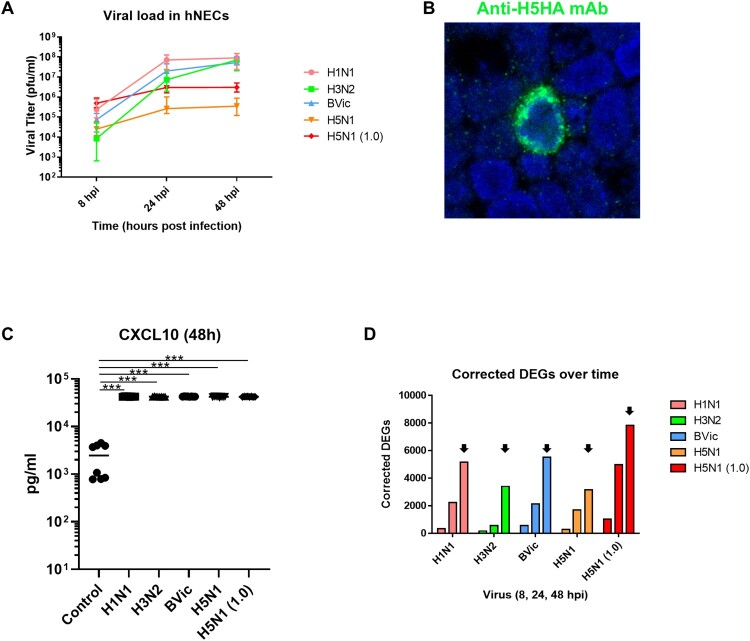
H5N1 Avian Influenza Infects the Human Nasal Epithelium with Lower Viral Replication and Elicits Comparable Host Responses. (A) Viral titre kinetics in hNECs infected with seasonal influenza viruses (at MOI of 0.1) and H5N1 virus (at MOI of 0.1 and 1.0). (B) Immunofluorescence staining with anti-H5 HA (green) in infected hNECs depicting active H5N1 infection of hNECs. (C) Secreted CXCL10 levels at 48 hpi in all influenza strains and control in hNECs infection. Statistical analysis was performed using 1-Way ANOVA with Tukey's correction for multiple comparison. *p < 0.05; **p < 0.01; ***p < 0.001. (D) Number of significantly differentially expressed genes (DEGs) in all seasonal versus avian influenza infections in hNECs. Black arrows indicate peak DEG numbers among all the time points tested (8, 24 and 48hpi).

We previously documented that our hNECs model is permissive to infection with multiple respiratory viruses, including influenza viruses. In this study, we compared hNEC infections between circulating community strains of seasonal influenza (H1N1, H3N2 and BVic), with a H5N1 HPAI strain isolated from a patient with severe disease. Upon infection of hNECs with seasonal and avian influenza viruses at MOI of 0.1, active infection was observed and infectious progeny viruses were detected as early as 8hpi (Figure 1(A)). In seasonal influenza infections, the infectious progeny titre increased at a similar rate, with a logarithmic increase between 8 and 24hpi, reaching the highest titre at 48hpi at around 10^7^ and 10^8^ PFU/mL. In contrast, while H5N1 virus was able to infect hNECs, the increase in infectious progeny exhibited a more linear trend, lacking the logarithmic increase phase. The highest titre of H5N1 peaked at around 10^5^ or 10^6^ PFU/mL, i.e. 1–2 Log_10_ lower than seasonal influenza viruses. Even with an elevated MOI of 1.0, avian influenza followed the same stunted increase in viral titre, suggesting less efficient replication in the upper airway. Despite its less-adapted infection, we confirmed that active H5N1 infection occurred in hNECs via staining of H5 HA in the infected hNECs (Figure 1(B)). Immunofluorescence co-staining further showed that the H5N1 clinical isolate infected the ciliated cells (positive for α- or βIV-tubulin) and goblet cells (positive for MUC5AC) in hNECs (Figure S1), whereas H5 HA co-staining was absent with the basal cell marker P63 (data not shown). We further validated these infected cell types by infection of hNECs with avian isolate of H5N1, which revealed consistent results (Figure S1). To verify active infection, our LUMINEX analyses of the apical supernatant at 48 hpi, showed significantly elevated CXCL10, a chemokine released in response to viral infection such as influenza (Figure 1(C)). Interestingly, although less adapted to infecting hNECs and producing lower viral titres, avian influenza infection of hNECs (at MOI of 1.0) yielded comparable numbers of DEGs when as seasonal influenza infections, especially at 48hpi, when the viral titres peaked for all infections (Figure 1(D)).

### H5N1 avian influenza triggers differential temporal pathway responses compared to seasonal influenza infection

**Figure 2.**
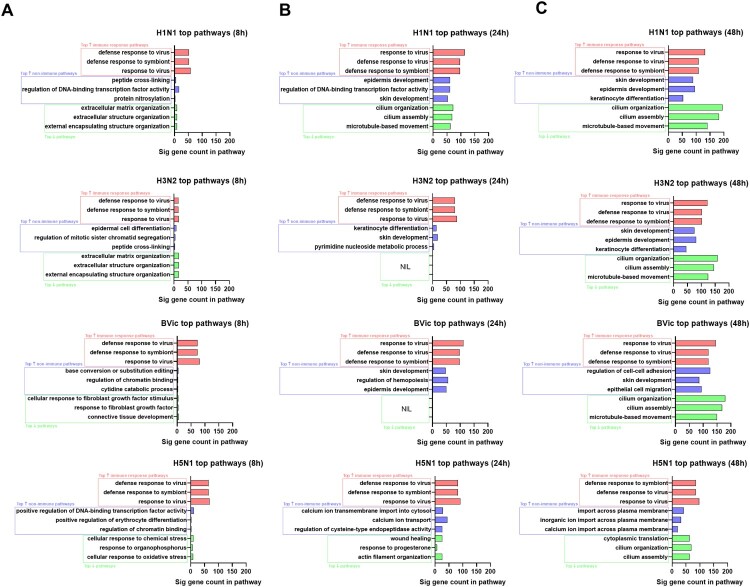
Gene Ontology Pathways of Infected in hNECs Showed Differential Upper Airway Responses Between Seasonal and Avian Influenza Infections. The top immune and non-immune pathways from up-regulated DEGs and top pathways from down-regulated DEGs at (A) 8hpi, (B) 24 hpi and (C) 48 hpi. Top up-regulated immune and non-immune pathways were determined by their functions in which immune pathways comprise antiviral immune response functions while non-immune pathways do not have direct function leading to initiation of antiviral immune response. The x-axis shows the number of significant DEGs that are present in the top pathways. Significant GO pathways were determined by hypergeometric analysis. Pathways with an adjusted *p*-value of <0.05 were considered significant.

We next analysed the DEGs obtained from RNAseq to perform a time course hypergeometric GO pathway analysis (Table S1-3). We identified the top-up-regulated pathways related to immune response and non-immune functions, as well as the top-down-regulated pathways at 8, 24 and 48hpi (Figure 2(A–C)). Early into infection (at 8 hpi), most of the significant pathways included innate immune functions up-regulated in response to influenza infections (Table 1). Comparatively, influenza B infection induced the greatest number of significant genes in the antiviral innate immune response pathways, while H3N2 influenza induced the least number. A small number of non-immune pathways were induced, mostly with similar molecular functions across all infections. On the other hand, down-regulated pathways in seasonal influenza infections were related to extracellular matrix, growth and differentiation, while down-regulated pathways in H5N1 influenza were involved in cellular stress responses. At the intermediate and late timepoints (24 and 48hpi), the significant pathways increasingly overlapped for seasonal influenza infections, with gradual increase of up-regulated innate antiviral response and developmental pathways (potentially involved in re-epithelization); as well as strong downregulation of ciliary pathways (Table 1). These changes indicated that the seasonal influenza responses in the upper airway epithelium followed a similar progression. Conversely, for H5N1 avian influenza, there was a slower increase in innate antiviral responses compared to seasonal influenza; while non-immune pathways revealed significant up-regulation of genes involved in ion transport pathways. The down-regulated H5N1 pathways were involved in ciliary functions as well as wound healing responses. Notably, the pathways associated with re-epithelization were absent in H5N1 influenza. Given the significant pathway changes were more consistent at the peak viral titre of 48hpi, this time-point was selected for subsequent analyses.

**Table 1.** Top GO Pathways (BP) in infection of hNECs (MOI of 0.1) with Seasonal versus Avian Influenza Strains at 8, 24 and 48 hpi.

H1N1 (8hpi)	H3N2 (8hpi)	BVic (8hpi)	H5N1 (8hpi)
**Top 10 Up-regulated Immune Response Pathways**			
defense response to virus	defense response to virus	defense response to virus	defense response to virus
defense response to symbiont	defense response to symbiont	defense response to symbiont	defense response to symbiont
response to virus	response to virus	response to virus	response to virus
negative regulation of viral process	negative regulation of viral genome replication	negative regulation of viral process	negative regulation of viral process
negative regulation of viral genome replication	regulation of viral genome replication	regulation of viral life cycle	negative regulation of viral genome replication
regulation of viral process	antiviral innate immune response	regulation of viral process	regulation of viral process
regulation of viral genome replication	viral genome replication	negative regulation of viral genome replication	regulation of viral life cycle
regulation of viral life cycle	negative regulation of viral process	regulation of viral genome replication	regulation of viral genome replication
viral genome replication	regulation of viral life cycle	regulation of innate immune response	viral genome replication
response to type I interferon	regulation of viral process	viral genome replication	interferon-mediated signaling pathway
**Top 10 Up-regulated Non-immune Pathways**			
peptide cross-linking	epidermal cell differentiation	base conversion or substitution editing	positive regulation of DNA-binding transcription factor activity
regulation of DNA-binding transcription factor activity	regulation of mitotic sister chromatid segregation	regulation of chromatin binding	positive regulation of erythrocyte differentiation
protein nitrosylation	peptide cross-linking	cytidine catabolic process	regulation of chromatin binding
peptidyl-cysteine S-nitrosylation	keratinocyte differentiation	cytidine deamination	positive regulation of tolerance induction
regulation of cysteine-type endopeptidase activity	epidermis development	cytidine to uridine editing	positive regulation of cysteine-type endopeptidase activity
negative regulation of proteolysis	skin development	cytidine metabolic process	positive regulation of release of sequestered calcium ion into cytosol
	mitotic spindle assembly checkpoint signaling	DNA deamination	
	spindle assembly checkpoint signaling	branching involved in labyrinthine layer morphogenesis	
	mitotic spindle checkpoint signaling	positive regulation of glial cell migration	
	negative regulation of chromosome organization	fibrinolysis	
**Top 10 Down-regulated Pathways**			
extracellular matrix organization	extracellular matrix organization	cellular response to fibroblast growth factor stimulus	cellular response to chemical stress
extracellular structure organization	extracellular structure organization	response to fibroblast growth factor	response to organophosphorus
external encapsulating structure organization	external encapsulating structure organization	connective tissue development	cellular response to oxidative stress
transforming growth factor beta production	cell-substrate adhesion	extracellular matrix organization	response to lipopolysaccharide
collagen metabolic process	regulation of cell-substrate adhesion	extracellular structure organization	response to purine-containing compound
endodermal cell differentiation	cell-matrix adhesion	external encapsulating structure organization	response to molecule of bacterial origin
endoderm formation	collagen fibril organization	cell growth	response to oxidative stress
formation of primary germ layer	integrin-mediated signaling pathway	icosanoid secretion	response to decreased oxygen levels
endoderm development	cellular response to amino acid stimulus	embryonic limb morphogenesis	positive regulation of miRNA metabolic process
ossification	cellular response to acid chemical	embryonic appendage morphogenesis	regulation of muscle contraction

H1N1 (24hpi)	H3N2 (24hpi)	BVic (24hpi)	H5N1 (24hpi)
**Top 10 Up-regulated Immune Response Pathways**			
response to virus	defense response to virus	response to virus	defense response to virus
defense response to virus	defense response to symbiont	defense response to virus	defense response to symbiont
defense response to symbiont	response to virus	defense response to symbiont	response to virus
regulation of innate immune response	negative regulation of viral process	regulation of innate immune response	negative regulation of viral process
positive regulation of defense response	regulation of innate immune response	negative regulation of viral process	regulation of viral life cycle
negative regulation of viral process	regulation of viral life cycle	positive regulation of defense response	negative regulation of viral genome replication
regulation of viral life cycle	regulation of viral process	regulation of viral life cycle	regulation of innate immune response
regulation of viral process	negative regulation of viral genome replication	response to molecule of bacterial origin	regulation of viral process
response to molecule of bacterial origin	regulation of viral genome replication	regulation of viral process	regulation of viral genome replication
positive regulation of cytokine production	interferon-mediated signaling pathway	positive regulation of cytokine production	positive regulation of cytokine production
**Top 10 Up-regulated Non-immune Pathways**			
epidermis development	keratinocyte differentiation	skin development	calcium ion transmembrane import into cytosol
regulation of DNA-binding transcription factor activity	skin development	regulation of haemopoeisis	calcium ion transport
skin development	pyrimidine nucleoside metabolic process	epidermis development	regulation of cysteine-type endopeptidase activity
regulation of cysteine-type endopeptidase activity	intermediate filament organization	response to endoplasmic reticulum stress	calcium ion import across plasma membrane
positive regulation of endopeptidase activity	NAD metabolic process	liver morphogenesis	regulation of monoatomic ion transmembrane transport
positive regulation of cysteine-type endopeptidase activity	nicotinamide nucleotide biosynthetic process	gland development	positive regulation of DNA-binding transcription factor activity
positive regulation of peptidase activity	pyridine nucleotide biosynthetic process	regulation of epithelial cell apoptotic process	calcium ion transmembrane transport
keratinocyte differentiation	positive regulation of proteolysis	response to hypoxia	import across plasma membrane
epidermal cell differentiation	wound healing	response to decreased oxygen levels	regulation of endopeptidase activity
regulation of cell-cell adhesion	pyridine-containing compound biosynthetic process	hepatocyte proliferation	regulation of DNA-binding transcription factor activity
**Top 10 Down-regulated Pathways**			
cilium organization			wound healing
cilium assembly			response to progesterone
microtubule-based movement			actin filament organization
cilium movement			response to ketone
cilium or flagellum-dependent cell motility			protein O-linked glycosylation via threonine
cilium-dependent cell motility			response to steroid hormone
epithelial cilium movement involved in extracellular fluid movement			response to radiation
microtubule-based transport			response to nutrient levels
cilium movement involved in cell motility			multi-organism reproductive process
extracellular transport			actin filament-based movement

H1N1 (48hpi)	H3N2 (48hpi)	BVic (48hpi)	H5N1 (48hpi)
**Top 10 Up-regulated Immune Response Pathways**			
response to virus	response to virus	response to virus	defense response to symbiont
defense response to virus	defense response to virus	defense response to virus	defense response to virus
defense response to symbiont	defense response to symbiont	defense response to symbiont	response to virus
positive regulation of cytokine production	regulation of innate immune response	positive regulation of cytokine production	negative regulation of viral process
response to lipopolysaccharide	positive regulation of cytokine production	positive regulation of defense response	regulation of viral life cycle
response to molecule of bacterial origin	positive regulation of defense response	regulation of innate immune response	regulation of innate immune response
regulation of innate immune response	response to molecule of bacterial origin	response to molecule of bacterial origin	regulation of viral process
positive regulation of defense response	response to lipopolysaccharide	response to lipopolysaccharide	negative regulation of viral genome replication
response to type II interferon	response to type II interferon	leukocyte migration	immune response-regulating signaling pathway
regulation of immune effector process	positive regulation of innate immune response	leukocyte cell-cell adhesion	immune response-activating signaling pathway
**Top 10 Up-regulated Non-immune Pathways**			
skin development	skin development	regulation of cell-cell adhesion	import across plasma membrane
epidermis development	epidermis development	skin development	inorganic cation import across plasma membrane
keratinocyte differentiation	keratinocyte differentiation	epithelial cell migration	inorganic ion import across plasma membrane
positive regulation of DNA-binding transcription factor activity	regulation of cell-cell adhesion	epithelium migration	calcium ion import across plasma membrane
regulation of endopeptidase activity	regulation of leukocyte cell-cell adhesion	epithelial cell proliferation	calcium ion transmembrane import into cytosol
regulation of DNA-binding transcription factor activity	epidermal cell differentiation	tissue migration	modulation of chemical synaptic transmission
positive regulation of cell-cell adhesion	positive regulation of cell-cell adhesion	regulation of haemopoiesis	antigen processing and presentation of exogenous peptide antigen via MHC class II
epidermal cell differentiation	regulation of cysteine-type endopeptidase activity	epidermis development	regulation of trans-synaptic signaling
positive regulation of proteolysis	positive regulation of DNA-binding transcription factor activity	endothelial cell migration	regulation of cell-cell adhesion
establishment of skin barrier	positive regulation of proteolysis	regulation of peptidase activity	regulation of membrane potential
**Top 10 Down-regulated Pathways**			
cilium organization	cilium organization	cilium organization	cytoplasmic translation
cilium assembly	cilium assembly	cilium assembly	cilium organization
microtubule-based movement	microtubule-based movement	microtubule-based movement	cilium assembly
cilium movement	cilium movement	cilium movement	microtubule-based movement
axoneme assembly	axoneme assembly	axoneme assembly	cilium movement
microtubule bundle formation	microtubule bundle formation	microtubule bundle formation	microtubule-based transport
intraciliary transport	cilium or flagellum-dependent cell motility	cilium or flagellum-dependent cell motility	axoneme assembly
microtubule-based transport	cilium-dependent cell motility	cilium-dependent cell motility	microtubule bundle formation
cilium or flagellum-dependent cell motility	microtubule-based transport	cilium movement involved in cell motility	ribosomal small subunit biogenesis
cilium-dependent cell motility	cilium movement involved in cell motility	microtubule-based transport	intraciliary transport

### Seasonal and avian influenza infection of hNECs stimulate differential host responses

**Figure 3.**
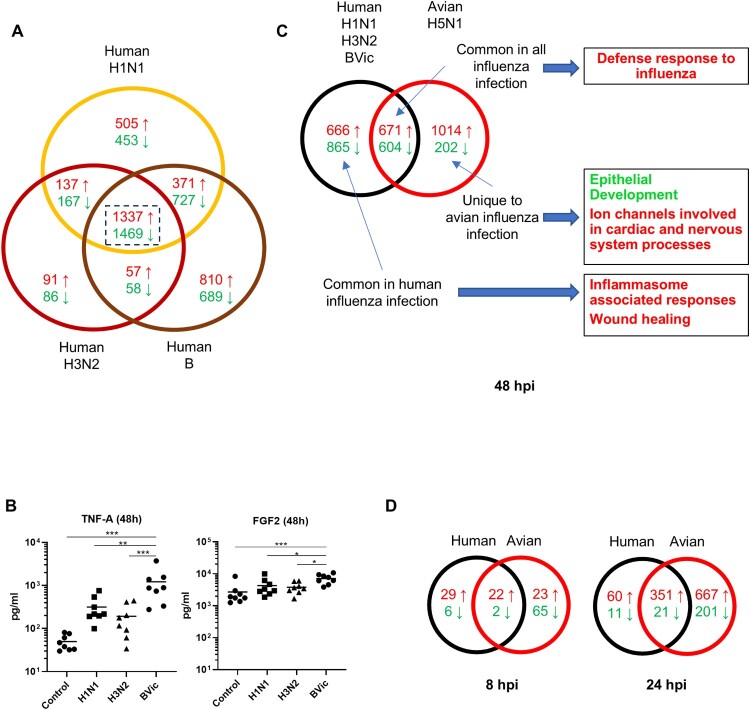
Human Seasonal Influenza Infections Exhibit Highly Similar Host Responses, While H5N1 Influenza Infection of hNECs Induce Unique Host Responses. (A) Venn diagram comparison of DEGs during human seasonal influenza infections of hNECs. The numbers indicate are significant DEGs that were up-regulated (red) or down-regulated (green). Influenza B induces the most DEGs among human seasonal influenza. (B) Comparison of TNFα and FGF2 protein levels in response to human seasonal influenza infection at 48hpi. Statistical analyses were performed using 1-Way ANOVA with Tukey's correction for multiple comparison. *p < 0.05; **p < 0.01; ***p < 0.001. (C) Overlapping DEGs in human influenza across all three strains were compared with DEGs from H5N1 avian influenza at 48hpi. Significant pathways of up or down-regulated DEGs (up-regulated – red; down-regulated – green) in the respective group (common, human only and avian only) were obtained via hypergeometric analysis. Pathways with an adjusted *p*-value of <0.05 were considered significant. (D) Venn diagrams for comparison of overlapping DEGs in between human seasonal influenza viruses and H5N1 avian influenza at 8 and 24hpi.

Compared to avian influenza, we have shown that seasonal influenza infections generally induced similar pathways. We further compared and contrasted the similarities and differences in DEGs and pathways between seasonal versus avian influenza. The common seasonal influenza DEGs were first established by identifying the DEGs overlapping between H1N1, H3N2 and BVic infections at 48hpi, the time-point with peak viral titres and responses. There was a large degree of overlap of genes between the three seasonal influenza infection tested, with the highest number of DEGs overlapping (Figure 3(A)). Among the seasonal influenza infections, BVic induced the greatest number of unique DEGs, and the highest responses among the overlapping genes. In addition, BVic infection also resulted in the highest levels of TNFα and FGF2 protein secretion at 48hpi (Figure 3(B)). We then further compared the overlapping seasonal influenza responses (1337 up-regulated; 1469 down-regulated DEGs) with those of H5N1 influenza infection. Only about one-third of total DEGs (671 up-regulated; 604 down-regulated DEGs) overlapped between seasonal and avian influenza infection of hNECs. The rest of the DEGs were unique to seasonal influenza and to H5N1 influenza (Figure 3(C)). Notably, avian influenza induced five times more unique up-regulated genes than down-regulated genes (1014 versus 202 DEGs) at 48hpi; whereas seasonal influenza induced relatively similar up and down-regulated DEGs. Among unique genes, this trend of H5N1 inducing more up-regulated than down-regulated DEGs was also observed at 24 hpi (Figure 3(D)). We also compared the percentage of DEGs overlapping with H5N1 influenza at higher MOI of 1.0, which revealed the highest percentage of overlap for H5N1 influenza at MOI of 0.1 but relatively lower overlaps for seasonal influenza infections at all time-points (Table S4). This observation is likely attributed to the different outcomes of infection of hNECs by seasonal versus avian influenza viruses. We then subjected three sets of DEGs from 48hpi (unique to seasonal, unique to H5N1, common in all influenza) to pathway analysis (Table S5). After analysing the significant pathways in GO-BP and REACTOME databases (Table S6), we then focused on pathways that displayed differential responses between seasonal and avian influenza at 48hpi, in order to elucidate potential expression patterns that could account for their differential pathology. Since there are largely overlapping pathways from both databases, most of the focused pathways are obtained from GO-BP, unless there are unique, non-overlapping pathways from the REACTOME database.

### The hNECs infected with avian influenza share common antiviral responses with seasonal influenza, but elicit relatively lower levels of expression

**Figure 4.**
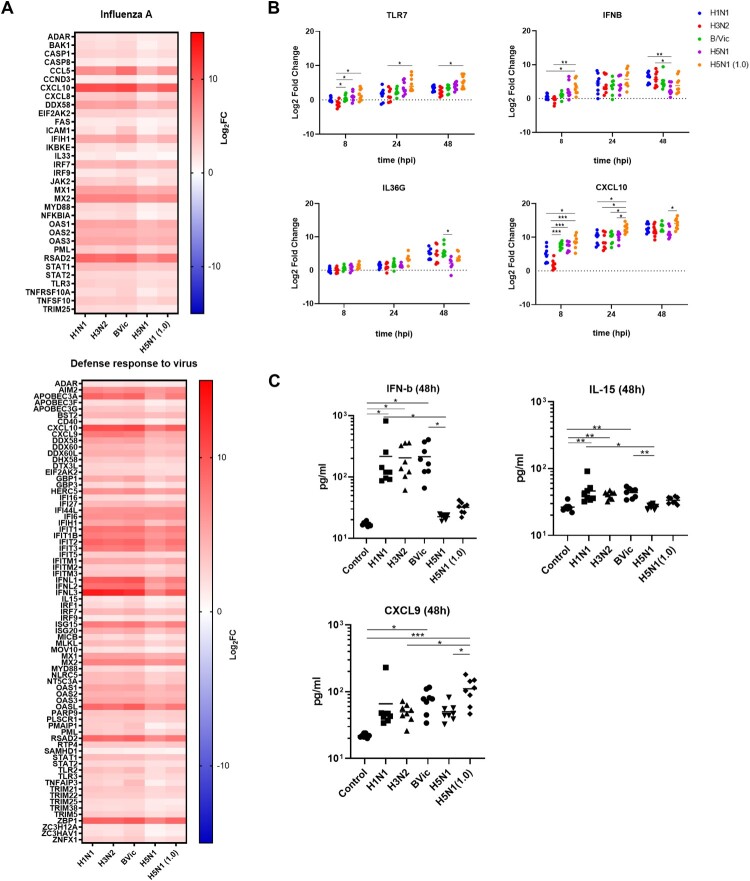
H5N1 Avian Influenza Infection of hNECs Induces Relatively Weaker Responses to Viruses. (A) Heatmaps of selected pathway common between human and avian influenza at 48hpi, mostly being response pathway to influenza and viruses. Gene expression value is presented in Log2FC. (B) qPCR validation of selected genes from significant pathway shared between human seasonal and avian influenza infection at 48hpi. Statistical analyses were conducted using 2-Way ANOVA with Tukey's correction for multiple comparison. (C) LUMINEX validation of apically secreted cytokines in significant pathways shared between human seasonal and avian influenza infection. Statistical analyses were performed using 1-Way ANOVA with Tukey's correction for multiple comparison. *p < 0.05; **p < 0.01; ***p < 0.001.

Heatmaps of the DEGs were generated to compare the overlapping and significantly enriched pathways for seasonal influenza versus H5N1 avian influenza responses. DEGs of anti-influenza A and virus defense responses were up-regulated for all influenza-infected in hNECs. Specifically, viral sensor, interferon, and type I immune cytokine and chemokine signalling pathways were all activated. However, the levels of activation of these response genes at 48 hpi were relatively lower in hNECs infected with H5N1 than seasonal influenza at MOI of 0.1 (Figure 4(A)). This finding is congruent with the individual pathway analysis, where H5N1 influenza (at MOI of 0.1) induced moderate early responses which did not strengthen as much over time, compared to seasonal influenza at the same MOIs. Interestingly, the magnitude of responses to H5N1 influenza at higher MOI of 1.0 rose to levels comparable with seasonal influenza at MOI of 0.1. We further validated this expression pattern using qPCR of selected genes in antiviral response pathways at 8, 24 and 48hpi. For H5N1 infection at lower MOI of 0.1, there was an earlier increase in expression of response genes (*TLR7*, *CXCL10*, *IFNB*, *IL36G*) at 8hpi – this eventually plateaued, either matching or overtaken by seasonal influenza expression responses at 48 hpi (Figure 4(B)). H5N1 influenza at a higher MOI of 1.0 did not lead to a stark increase in anti-influenza responses at all time points. Interestingly, compared to seasonal influenza, our LUMINEX analysis showed that there was significantly lower apical secretion of IFNβ and IL-15 in response to H5N1 influenza at MOI of 0.1, although CXCL9 secretion was elevated at H5N1 MOI of 1.0 (Figure 4(C)). There were also other secretory factors whose levels were unchanged, or non-significantly altered by influenza infection (Figure S2).

### Avian influenza infection of hNECs induces weaker inflammasome activation

**Figure 5.**
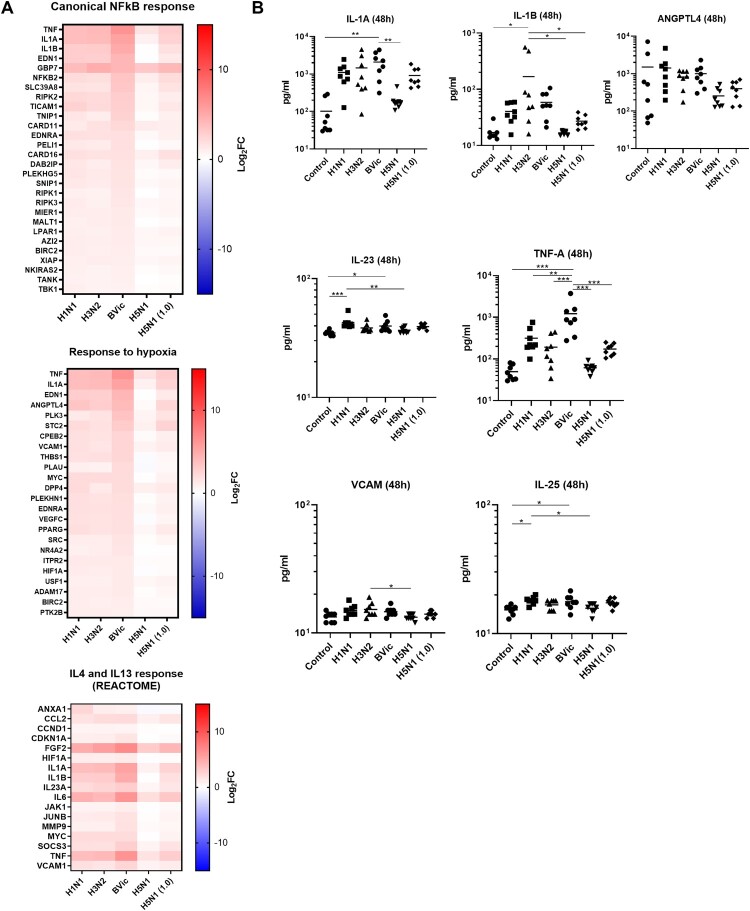
H5N1 Avian Influenza Infection of hNECs Elicit Subdued Inflammasome Activation Compared to Human Seasonal Influenza. (A) Heatmaps of selected pathway significantly up-regulated in human seasonal influenza at 48hpi, including inflammasome, NFκB and hypoxic responses. Gene expression value is presented in Log2FC. (B) LUMINEX validation of apically secreted cytokines from significant pathways only found in human seasonal influenza infection at 48hpi. Statistical analyses were performed using 1-Way ANOVA with Tukey's correction for multiple comparison. *p < 0.05; **p < 0.01; ***p < 0.001.

Analysis of DEGs identified pathways mediating responses to canonical NFκB, hypoxia, IL4 and IL13 (Reactome pathway) common to seasonal influenza infections (at MOI of 0.1). These pathways are involved in inflammasome activation in infected epithelial cells, as part of the early immune response against viruses (Figure 5(A)). This finding suggests that while the upper airway can be infected by avian influenza virus, the upper airway does not induce a full repertoire of responses to facilitate its clearance (compared to seasonal influenza viruses). Even at higher MOI of 1.0 of H5N1 infection, these levels of gene expression remained lower than those for seasonal influenza. Further, our LUMINEX analysis of apically secreted cytokines for H5N1 influenza also reinforced that the inflammasome-associated cytokines were indeed not strongly secreted at the protein level. At 48hpi with H5N1 virus at MOI of 0.1, we observed that IL-1α, IL-1β, TNFα and ANGPTL4 were secreted at low levels which were comparable to the time-matched uninfected control. Overall, the secretion levels of inflammasome factors in H5N1 infection (even at higher MOI of 1.0) were generally lower compared to seasonal influenza (e.g. significantly higher levels of IL-1α in BVic; and IL-1β in H3N2) (Figure 5(B)). The secretion levels of IL-23, VCAM1 and IL-25 (an epithelial alarmin) were relatively lower for H5N1 influenza compared to seasonal influenza (e.g. higher levels of IL-23 in H1N1 and VCAM in H3N2 infection) (Figure 5(B)). These observations highlight the differences between inflammatory responses elicited by seasonal versus avian influenza.

### Avian influenza infection of hNECs suppresses epithelial development genes and modulates stress responses

**Figure 6.**
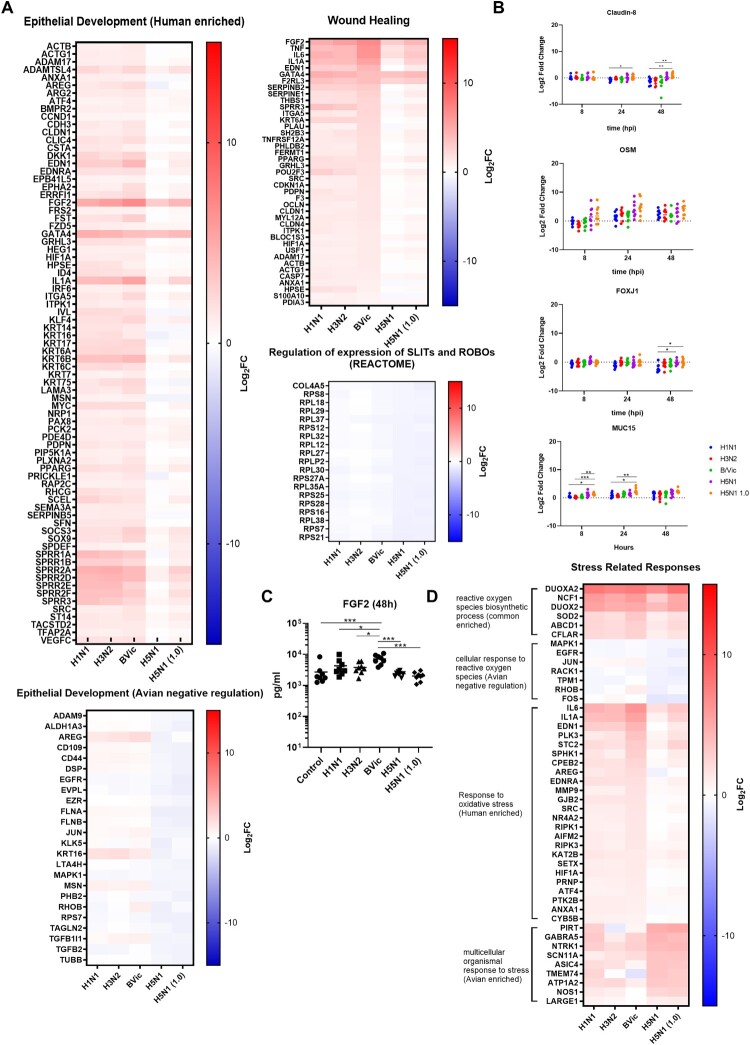
H5N1 Avian Influenza Infection of hNECs Differentially Downregulates Epithelial Development and Wound Healing Genes. (A) Heatmaps of selected pathways significantly up-regulated in human influenza but down-regulated in avian influenza at 48hpi, including regulation of epithelial development, wound healing and SLIT/ROBO pathways. Gene expression value is presented in Log2FC. (B) qPCR validation of selected genes from significant pathways differentially up-regulated in human seasonal influenza, while down-regulated in avian influenza infection at 48hpi. Statistical analyses were conducted using 2-Way ANOVA with Tukey's correction for multiple comparison. (C) LUMINEX validation of apically secreted FGF2 identified from significant pathway up-regulated in human seasonal influenza infection at 48hpi. (D) Heatmaps of stress related responses at 48hpi showing differences between human seasonal and avian influenza infections. Gene expression value is presented in Log2FC. Statistical analyses were performed using 1-Way ANOVA with Tukey's correction for multiple comparison *p < 0.05; **p < 0.01; ***p < 0.001.

We previously observed that hNECs can undergo re-epithelization during *in vitro* infection to repair damage to the epithelial barrier [29,30]. In all influenza infections, we indeed observed common down-regulated pathways pertaining to ciliary development and function, suggesting loss or reduction of cilia (Figure 2, Table S6), which may impair the upper airway's ability to protect against the pathogen. Interestingly, in response to injury, only seasonal influenza resulted in the enrichment of epithelial development genes, at 48hpi (Figure 6(A)). This altered epithelial development involved DEGs that regulate hypoxia, wound healing, and keratinization. Thus, genes with protective or repair functions may be up-regulated for re-epithelization during seasonal influenza infection. In contrast, with H5N1 influenza, genes in these pathways were not expressed strongly or significantly. Furthermore, in response to H5N1 influenza at 48 hpi, we identified a unique set of down-regulated genes from the epithelial development pathway and SLIT/ROBO regulatory pathway, suggestive of compromised epithelial barrier integrity and suppression of re-epithelization. Further validation by qPCR showed some alteration in genes involved in epithelial integrity, i.e. trend of increased expression of tight junction marker *CLAUDIN-8* and ciliary marker *FOXJ* in avian influenza (particularly at higher MOI) over the time-course of 8, 24 and 48 hpi (Figure 6(B)). Importantly, we also observed more rapid expression of *OSM* gene, a factor involved in epithelial barrier disruption (albeit non-significantly) early in avian influenza infection (Figure 6(B)). Other changes include up-regulated expression of membrane-tethered mucin, such as MUC15 in influenza infection [31]. *MUC15* exhibited higher mRNA expression at 8 and 24hpi, especially with H5N1 infection at MOI of 1.0. In addition, apically-secreted FGF2 (an early factor of re-epithelization) was only elevated in seasonal but not avian influenza infection of hNECs at 48hpi, based on our LUMINEX analysis of apically secreted cytokines (Figure 6(C)). Levels of other secretory factors and gene transcripts are shown in Figures S2 and S3. Additional pathway analyses indicate that the differences in damage and re-epithelization during influenza infection may be attributed to altered stress responses in infected host cells. Enrichment of reactive oxygen species processes was observed in both seasonal and H5N1 influenza infection. However, only seasonal influenza exhibited enrichment in oxidative stress responses, whereas H5N1 infection triggered multi-cellular organismal responses to stress (Figure 6(D)).

### Avian influenza infection of hNECs strongly induces ion-channel and ion-transport proteins

**Figure 7.**
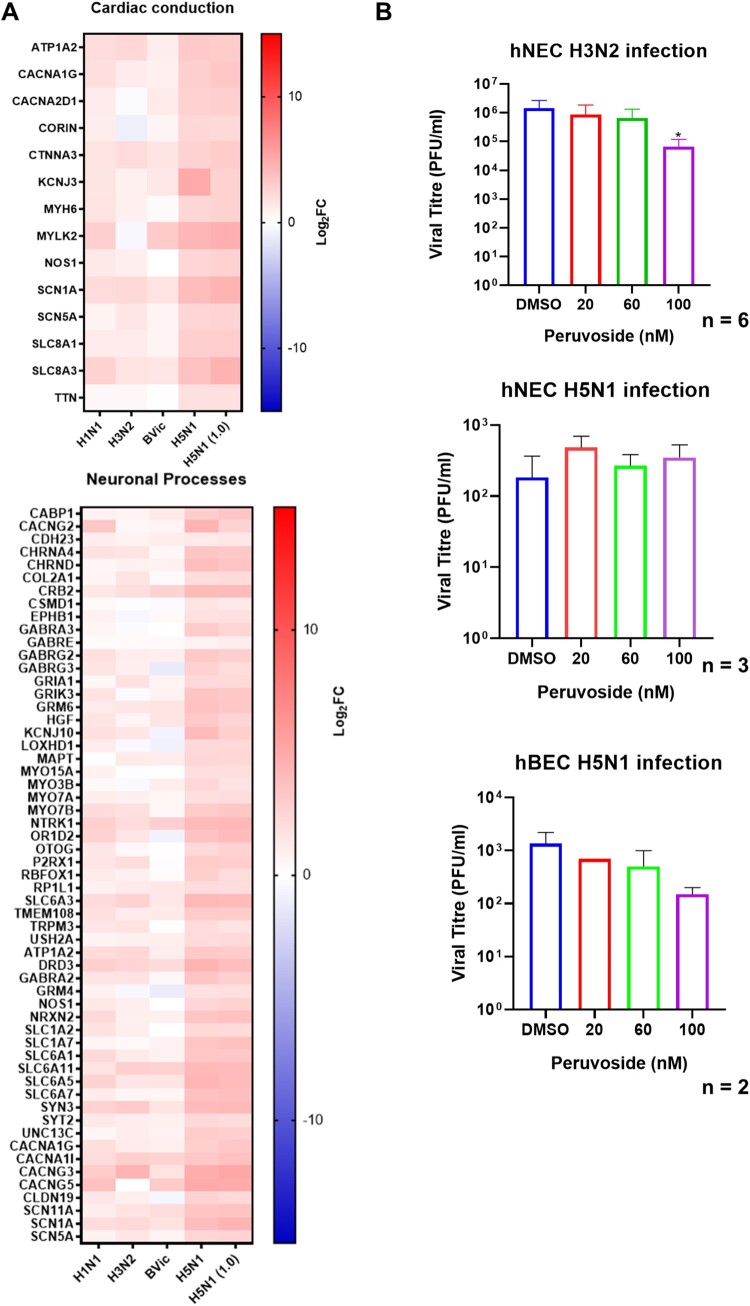
Avian (H5N1) Infection Resulted in Cardiac and Neuronal Pathway Enrichment. (A) Heatmaps of selected pathways significantly up-regulated in avian influenza at 48hpi, including cardiac conduction and neuronal processes pathways which are enriched with ion transport genes. Gene expression value is presented in Log2FC. (B) The hNECs and/or hBECs infected with H3N2 and H5N1 were subjected to treatment with peruvoside, a modulator of cellular calcium ions, at concentrations of 20, 60 and 100nM. At 100nM, peruvoside significantly reduced the H3N2 viral titre hNECs, and of H5N1 viral titre infected hBECs. Statistical analyses were conducted using 1-Way ANOVA with Dunnett's correction for multiple comparison. *p < 0.05.

When analysing the unique avian influenza DEGs, we observed many enriched pathways related to cardiac conduction and neuronal processes at 48hpi (Figure 7(A)). The DEGs in these enriched pathways induced in response to H5N1 infection were identified as genes involved in ion transport. The stronger expression of these ion transport genes during H5N1 influenza infection may allude to potentially druggable pathways against avian influenza. To test this hypothesis, we treated infected hNECs with peruvoside, an ion transport modulator that inhibits the sodium/potassium pump to increase intracellular calcium ions. Peruvoside was selected since our pathway analysis revealed notable enrichment of calcium ion transport during H5N1 infection of hNECs. This treatment would assess how modulation of ion channels would affect influenza virus replication. Infections with different strains of seasonal (H3N2, A/Aichi/2/1968) and avian (H5N1, A/mallard/Wisconsin/2576/2009) influenza were performed to evaluate the impact of ion channel modulation. Interestingly, treatment of infected hNECs with peruvoside (at 100nM) exerted an inhibitory effect on H3N2 but not on H5N1 influenza at 48 hpi (Figure 7(B)). However, peruvoside treatment at 100nM exerted an inhibitory trend on H5N1 replication in hBECs (Figure 7(B)), which exhibited different viral titre compared to hNECs infection (Figure S4).

## Discussion

Influenza virus is one of the most successful respiratory viruses circulating among the human population, in addition to a multitude of avian and mammalian species that facilitate reassortment and emergence of novel influenza strains with pandemic potential [5,32]. Hence, a robust system that enables comparison of different emerging influenza strains is highly desirable, as a means to rapidly ascertain virus infectivity of the human nasal epithelium and to predict their transmission capacity and disease severity [33]. To accomplish this, we previously established influenza infection models using *in vitro* ALI cultures of hNECs to investigate transcriptomes following H3N2 infection [14,15,34]. The model of hNECs facilitates the study of the initial contact with both avian and seasonal influenza viruses to elucidate their unique interactions and responses. This is particularly crucial for HPAI (such as H5N1 or H7N9) where understanding of the upper airway responses is lacking, since many studies focus on the lower airway which is more susceptible to avian influenza. This study expanded the relevance of this model by comparing the transcriptomic profiles in response to infection of the upper airway with H5N1 HPAI virus (isolated from a patient with severe disease) versus representative clinical isolates of human seasonal influenza strains (H1N1, H3N2, BVic). The current consensus is that avian influenza viruses are more adapted to infecting the lower airway epithelium of humans, due to similarities of virus receptors to avian hosts. Generally, avian influenza viruses only infect humans who are exposed to high challenge doses of virus. This study sought to differentiate avian versus seasonal influenza virus interactions with the upper airway of the human host, as well as the responses that contribute to the differential pathogenesis.

Firstly, harnessing hNECs would be able to quickly evaluate the infectivity of the virus and thus predict its probability for widespread transmission [33,35]. ALI cultures of airway epithelial cells have been applied successfully in the initial understanding of SARS-CoV-2 infectivity and immune responses [27,36]. The ability of influenza virus to efficiently infect and replicate in an upper airway model system correlates with strong viral shedding and transmissibility. In our study, we found that all strains of human influenza viruses tested were highly adapted to infecting hNECs. On the other hand, avian influenza virus resulted in less efficient hNECs infection, irrespective of the infective dose or MOI. We further showed that avian influenza virus infects the ciliated cells and goblet cells, located apically in the hNECs culture, which is similar to seasonal influenza infection [14,37]. This indicates that H5N1 virus is able to target and replicate in the same cell types of the upper airway (albeit less efficiently), implying the possibility of human-to-human transmission. Therefore, precautions should be adopted in clinical and hospital settings where there may be high concentrations of virus leading to transmission among persons in close proximity, as evidenced by aerosol spread of influenza and SARS-CoV-2 [22,38,39].

Once the infectivity of a specific influenza strain in the hNECs model is established, the initial immune responses and virus interactions with the nasal epithelium can be elucidated, including subsequent pathways that lead to severe lower airway infections. Although common antiviral transcriptomic responses between seasonal and avian influenza were evident, there was a distinct difference where H5N1 induced relatively lower anti-influenza responses compared to seasonal influenza. While this may be explained by the lower adaptability of H5N1 to infect hNECs, raising the infective dose of H5N1 by ten-fold still did not result in markedly elevated responses. Therefore, this may be explained by other factors, such as differences in the interferon antagonism and evasion strategies of avian versus seasonal influenza viral proteins. A possible mechanism for the differential induction of antiviral responses by interferon antagonism is via the PB1-F2 protein, which is absent in influenza B virus whose infection induced the strongest responses [40]. The PB1-F2 protein of avian influenza A has additional functions compared to human influenza A [41,42]. In addition, certain avian influenza virus mutations also enhances its virulence and explain its differential pathogenesis [9]. The relatively lower peak titre and shedding of H5N1 virus may account for its relatively weaker activation of influenza responses in hNECs, which may in turn delay viral clearance and promote viral dissemination to the lower airway. Another crucial distinction between avian and seasonal influenza infection of hNECs pertains to their differences in expression of the immune repertoire (e.g. inflammasome-activating genes) and secretion of immune mediators. While inflammasome over-activation in the lower airway is a major contributor to severe pathology of HPAI [43], the subdued inflammasome activation in the upper airway may be a unique feature of avian influenza infections. The levels of inflammasome factor activation in seasonal influenza may serve a protective function that constrains infection to the upper airway[44]. The comparison of inflammasome activation levels in influenza infections of varying severity thus warrants future in-depth studies, including validation of mechanism such as detection of ASC granules [45]. Such insights may help to design novel strategies to mitigate lower airway injury.

The first-line defense of the mucociliary barrier of the upper airway is vital in protecting the lower airway via mechanical and immunological clearance of incoming pathogens and insults [46,47]. Influenza virus can cause epithelial destruction and cell death that compromises the integrity of the muco-ciliary barrier [14,48,49], which can aid virus dissemination to the lower airway that culminates in more severe disease. We observed down-regulation of ciliary genes in all infections, likely indicative of mucociliary barrier destruction. While seasonal influenza infection induced expression of genes mediating epithelial development and wound healing to facilitate re-epithelization, these gene enrichments were not noted for H5N1 infection. In H5N1 influenza, the down-regulated DEGs in the SLIT/ROBO pathway, which is important in preventing vascular leakage, may further compromise the epithelial barrier and facilitate diffuse inflammation and cytokine storm [50,51]. Finally, diminished responses to oxidative stress in H5N1 influenza may also impair re-epithelization and repair of the airway epithelium [52]. Taken together, the compromised mucociliary barrier, suppression of re-epithelization and dysregulated vascular leakage likely contribute to viral dissemination and injury that ultimately leads to severe H5N1 infections.

Influenza virus infection can perturb or interact with epithelial ion channels (including calcium, potassium and other channels) which may promote virus entry, augment airway epithelial cell secretion, and lead to pulmonary oedema [53–55]. In our study, Influenza infection does indeed modulate ion transport functions [56], where we revealed stronger upregulation of ion and calcium transport genes and pathways (related to cardiac and neuronal processes) in avian compared to seasonal influenza infection of hNECs. Peruvoside is an ion transport-modulating drug that alters intracellular calcium, and shows promise in inhibiting influenza infection [57]. Interestingly, we found that, peruvoside inhibited replication of seasonal influenza virus in hNECs; and dampened H5N1 virus in hBECs. This may be due to differences in the kinetics of seasonal and avian influenza virus replication in hNECs compared to hBECs [58], or potentially due to stronger calcium channel expression and calcium modulation in hBECs that allows more effective targeting by peruvoside. Thus, the inhibition of influenza virus replication by peruvoside suggests that the modulation of ion transport could be further explored as a potential treatment strategy against influenza. Furthermore, the enrichment of these pathways potentially indicates non-respiratory involvement of H5N1 avian influenza. Avian and other influenza viruses are able to infect neuronal cells *in vitro* and *in vivo*, and are also associated with cardiac injury in hospitalized patients [59,60]. It would be interesting to investigate whether H5N1 infection of primary human neuronal and cardiac cells could dysregulate specific ion channels, contributing to its neurologic and cardiac pathology [61,62].

This study does have its limitations. Firstly, we only performed infection with H5N1 virus whose findings may not be generalized to viral pathogenesis associated with other avian influenza subtypes such as H7N9. Cell death was also observed in some of the infections at 48 hpi, which precluded infection experiments beyond the time-point. Moreover, certain differences in host response pathways may also arise from disparities in adaptation and viral titre between seasonal and avian influenza infection (which lacks a logarithmic increase in titre) of hNECs. However, despite similar viral titres at 8 hpi, the pathways activated in response to seasonal and avian influenza exhibited divergence. Furthermore, when comparing the percentage of overlapping DEGs over time with H5N1 infection at MOI of 1.0, the highest percentage overlap was with H5N1 infection at MOI of 0.1, reflecting convergence of the H5N1 response pathway. These findings have elucidated the differential responses of hNECs to seasonal versus avian influenza, which can explain the key differences in their pathogenesis. The identification of these molecular patterns and factors in the upper airway can advance our understanding of severe avian influenza infection in humans, and potentially enhance diagnostic preventive and management strategies against HPAI infection in humans. These findings can also be compared and contrasted against the responses in hNECs infected with the more recent clade 2.3.4.4b H5N1 strain in cattle, to observe if any divergence of responses were present in a mammalian H5N1 [13,63]. Such studies may provide valuable information for predicting the potential pathogenic responses, mammalian and human transmissibility of future H5N1 and other avian influenza virus strains.

## Conclusion

The physiologically relevant model of hNECs has been successfully harnessed to investigate host adaptation and responses between human seasonal influenza versus and avian influenza virus infections in the human upper airway. This comparative analysis has elucidated unique responses and pathways of H5N1 infection which are distinct from those of seasonal influenza infection of hNECs. Although H5N1 replication in human upper airway cells is less efficient, there remains the risk of transmission of avian influenza viruses to susceptible humans. Therefore, it is vital to compare human nasal epithelial responses to infection with other avian influenza strains, particularly the more recent bovine H5N1 strain that has already caused human infections.

## Supplementary Material

Table S6 Full Pathway List of Human Seasonal versus Avian Influenza.xlsx

Table S5 List of Genes for Comparison of Human Seasonal and Avian Influenza.xlsx

Figure S1.TIF

Figure S2.TIF

Table S4 Percentage of Overlapping DEGs Between Human Seasonal and Avian Influenza.xlsx

Figure S4.TIF

Table S3 Full Pathway List at 48 hpi.xlsx

Figure S3.TIF

Table S2 Full Pathway List at 24 hpi.xlsx

Table S1 Full Pathway List at 8 hpi.xlsx
