# Supplementary figures and images for: Unique immune and other responses of human nasal epithelial cells infected with H5N1 avian influenza virus compared to seasonal human influenza A and B viruses
Source: Emerg Microbes Infect. 2025 Mar 24;14(1):2484330. doi: 10.1080/22221751.2025.2484330 (PMC11980200; doi:10.1080/22221751.2025.2484330)

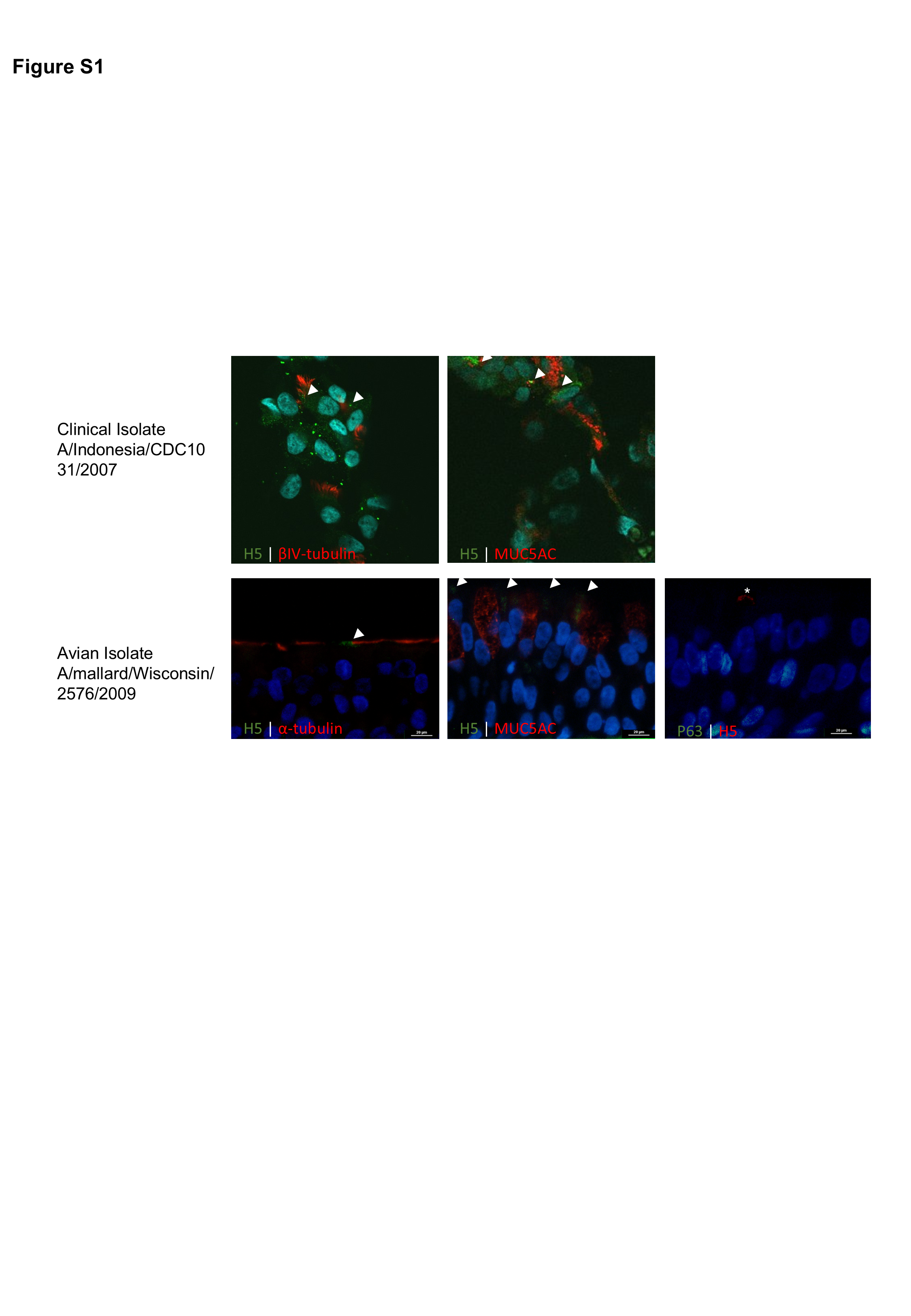

Supplement: Figure S1.TIF [file TEMI_A_2484330_SM9107.tif]

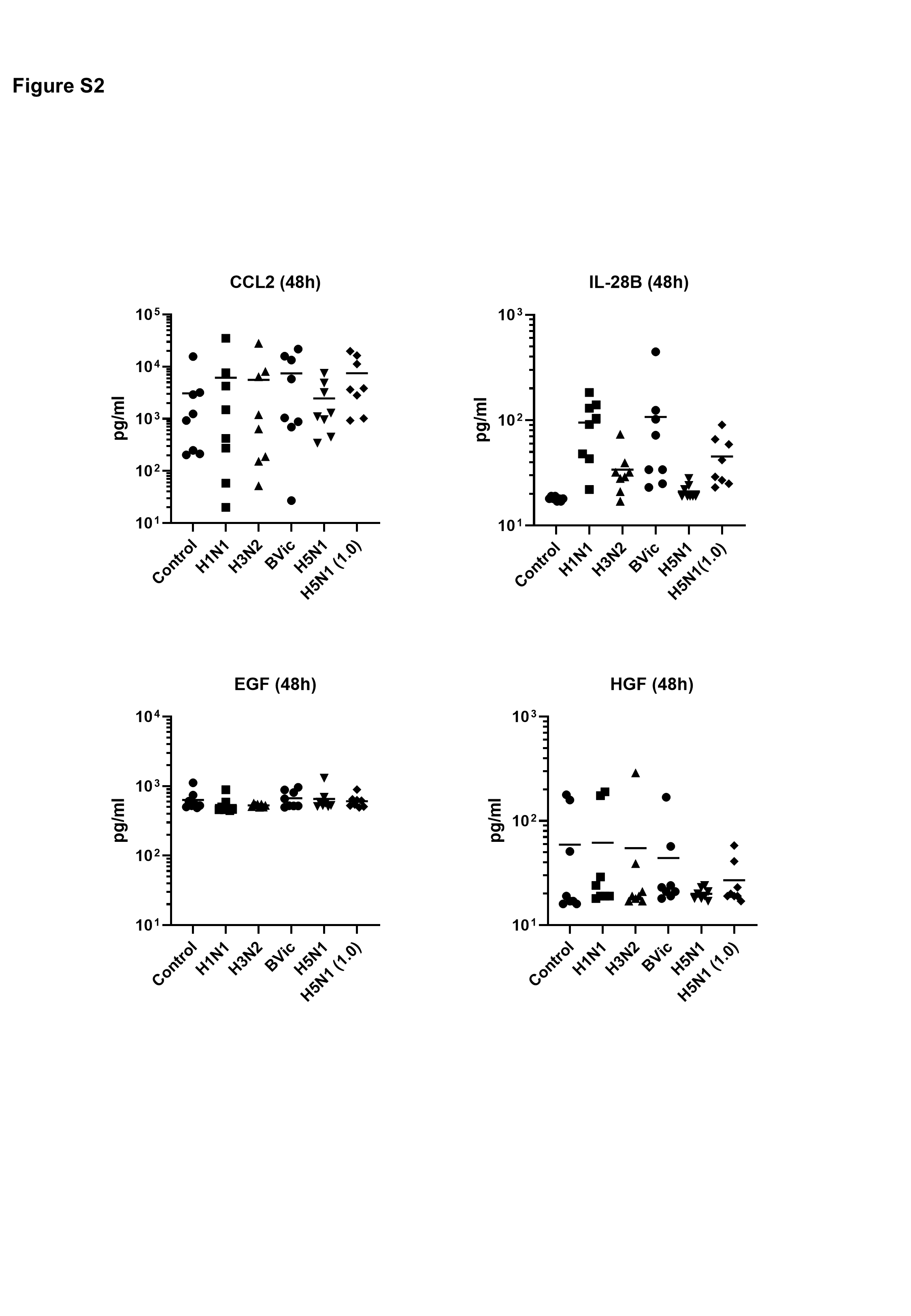

Supplement: Figure S2.TIF [file TEMI_A_2484330_SM9106.tif]

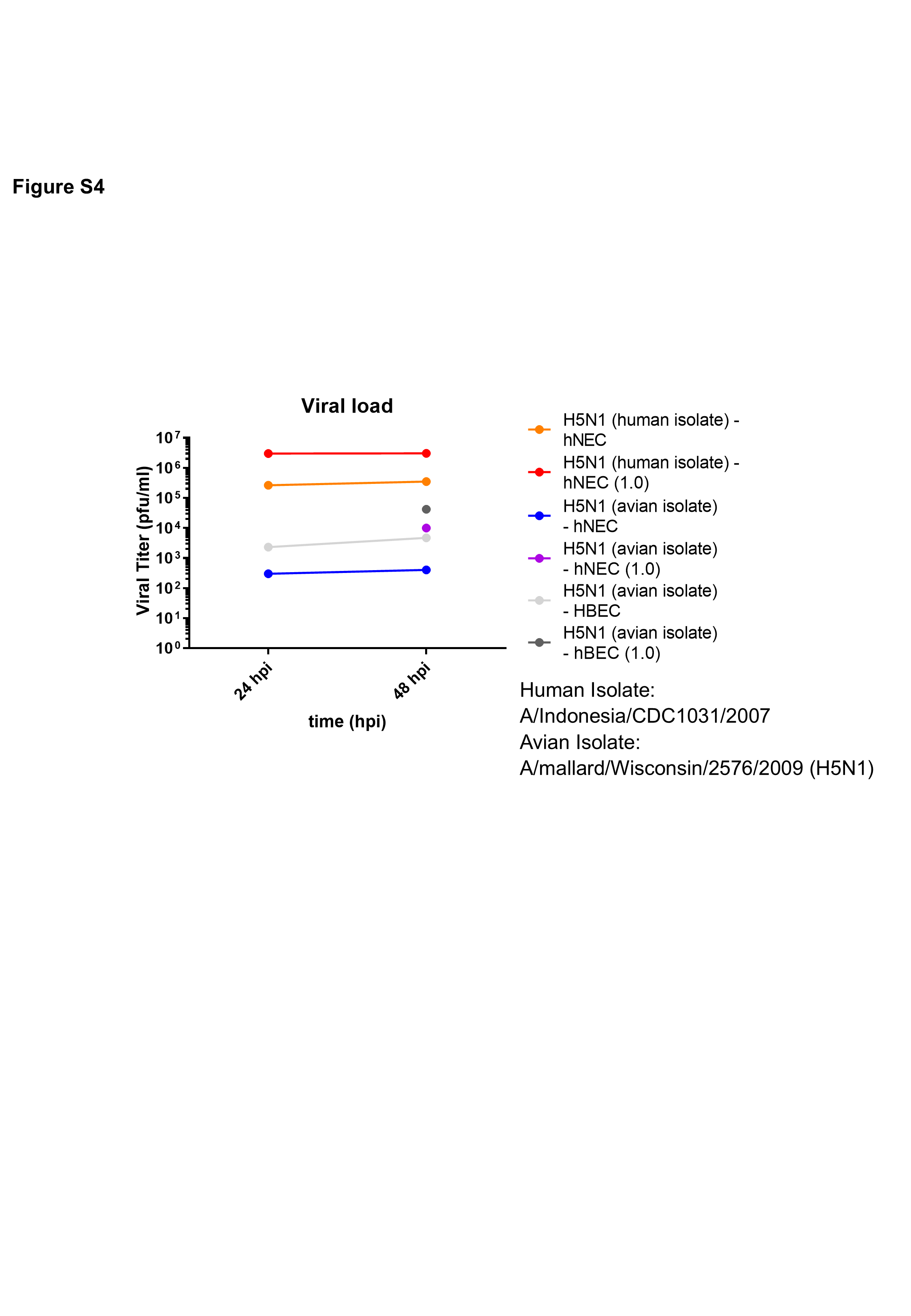

Supplement: Figure S4.TIF [file TEMI_A_2484330_SM9104.tif]

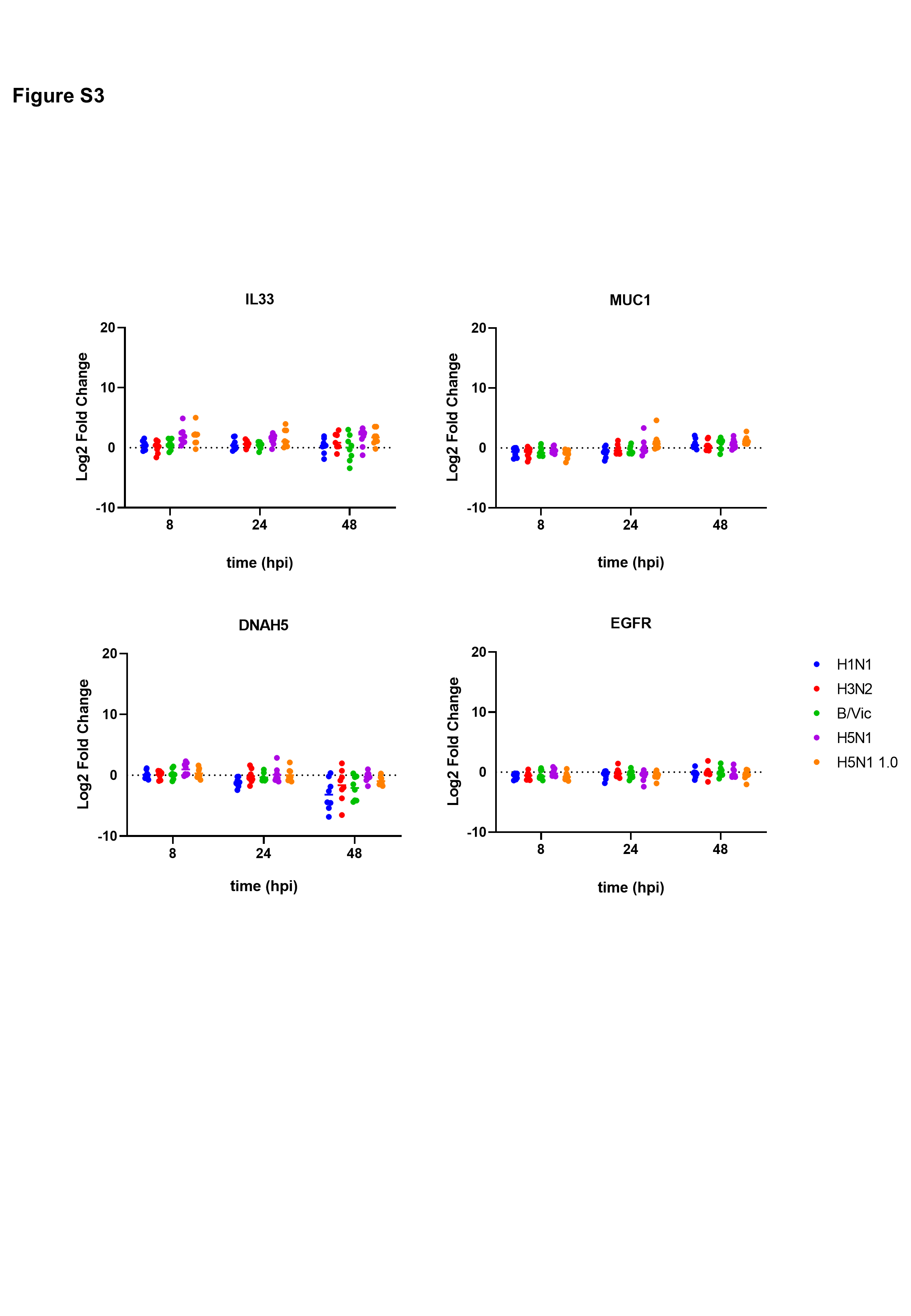

Supplement: Figure S3.TIF [file TEMI_A_2484330_SM9102.tif]
